# Antidepressants and Mood Stabilizers: Novel Research Avenues and Clinical Insights for Bipolar Depression

**DOI:** 10.3390/ijms18112406

**Published:** 2017-11-13

**Authors:** In Hee Shim, Young Sup Woo, Moon-Doo Kim, Won-Myong Bahk

**Affiliations:** 1 Department of Psychiatry, Cancer Center, Dongnam Institute of Radiological & Medical Sciences, Busan 46033, Korea; ihshim1224@gmail.com; 2 Department of Psychiatry, College of Medicine, The Catholic University of Korea, Seoul 07345, Korea; youngwoo@catholic.ac.kr; 3 Department of Psychiatry, School of Medicine, Jeju National University, Jeju 63241, Korea; mdkim66@jejunu.ac.kr

**Keywords:** antidepressant, mood-stabilizer, lithium, depression, mixed features, major depressive episode, bipolar disorder

## Abstract

The concept of the bipolar-spectrum and of mixed features being a bridge between major depressive disorders and bipolar disorders (BDs) has become increasingly important in mood-disorder diagnoses. Under these circumstances, antidepressants (ADs) and mood stabilizers (MSs) should be used with caution in the treatment of major depressive episodes (MDEs) and to obtain long-term stability in BDs. Before treating MDEs, screening tools, specific symptom evaluation and medical history should be used to distinguish between bipolarity and mixed features in patients for whom AD monotherapy may present a risk. In these patients, a combination of ADs plus MSs or atypical antipsychotics is recommended, rather than AD monotherapy. Studies evaluating MSs for bipolar depression suggest that lamotrigine is the most reliable treatment and lithium has modest effects; there is a lack of clear evidence regarding the efficacy of valproate and carbamazepine. Recently, significant progress has been made with respect to the pathophysiology of mood disorders and the application of potential biomarkers. There is an opportunity to study novel drug mechanisms through the rediscovery of fast-acting drugs such as ketamine. It is anticipated that future research developments will involve the discovery of potential targets for new drugs and their application to personalized treatments.

## 1. Introduction

Recent preliminary evidence suggests a potential trans-nosological effect of selected atypical antipsychotics (AAPs) beyond only psychotic disorders. Treatment with antipsychotics is an effective augmentation option for major depressive disorders (MDDs), bipolar mania and depression. For cases of non-psychotic depression with no response to the initial treatment strategy, some guidelines suggest augmentation of treatment with an AAP such as aripiprazole or quetiapine [1,2,3]. In the past, the preferred initial treatment strategy for mania, regardless of type, was MSs plus AAPs; however, recently, AAP monotherapy has been considered the most effective first-line treatment [4,5,6]. AAP monotherapy is recommended for mildly severe bipolar depression, while AAPs combined with mood stabilizers (MSs) or antidepressants (ADs) are recommended for moderate to severe depression [4,7].

Despite the increasing use of AAPs, MSs continue to play an important role in the management of bipolar mania and depression. Most mood disorders are characterized by the onset of a major depressive episode (MDE), and AD monotherapy is typically the first-line treatment. However, for bipolar disorders (BDs), AD monotherapy is very rarely recommended despite the high prevalence of depressive episodes. The risk-benefit profile of AD monotherapy for BDs remains controversial. Most guidelines suggest ADs as an adjuvant therapy with MSs [8], as a short-term rather than long-term treatment [9]. The proper use of MSs with ADs is very important during the acute phase of MDEs and the maintenance phase of BDs.

This review provides an overview of standard AD and MS treatments for both the acute phase of MDEs and long-term stabilization of BDs.

## 2. Comparison of MDEs in MDDs and BDs

For MDEs, it is difficult to distinguish between MDDs and BDs based on the clinical symptoms alone. The Diagnostic and Statistical Manual of Mental Disorder (DSM) and the International Classification of Disease diagnostic criteria for the two disorders are very similar. In research and in clinical practice, assessments of symptom severity and changes in symptoms are conducted using the same methods (e.g., the Hamilton Depression Rating Scale (HAMD) or the Montgomery-Asberg Depression Rating Scale (MADRS)). Because most patients with BDs present with the onset of a MDE, one-half to two-thirds of patients are initially misdiagnosed [10,11]. More than one-third remain misdiagnosed for 5–10 years or more [12,13].

Although there are many similarities between unipolar and bipolar depression, there are a number of significant differences. Patients with MDDs typically report more somatization and affective symptoms such as anxiety, anger, and agitation, and less anhedonia [14]. Contrarily, patients with bipolar depression tend to experience more atypical symptoms such as hypersomnia, hyperphagia, and leaden paralysis, as well as psychomotor retardation, psychotic features, pathological guilt, and lability of mood, although there are no pathognomonic biomarkers or clinical characteristics [15]. Regarding the long-term course of these illnesses, patients with bipolar depression are more likely to have an earlier age of onset [16], more prior episodes of depression [17], shorter depressive episodes [18], a family history of BDs [19], comorbid substance use disorder [20], and a higher risk of postpartum depression [21].

**Figure 1:**
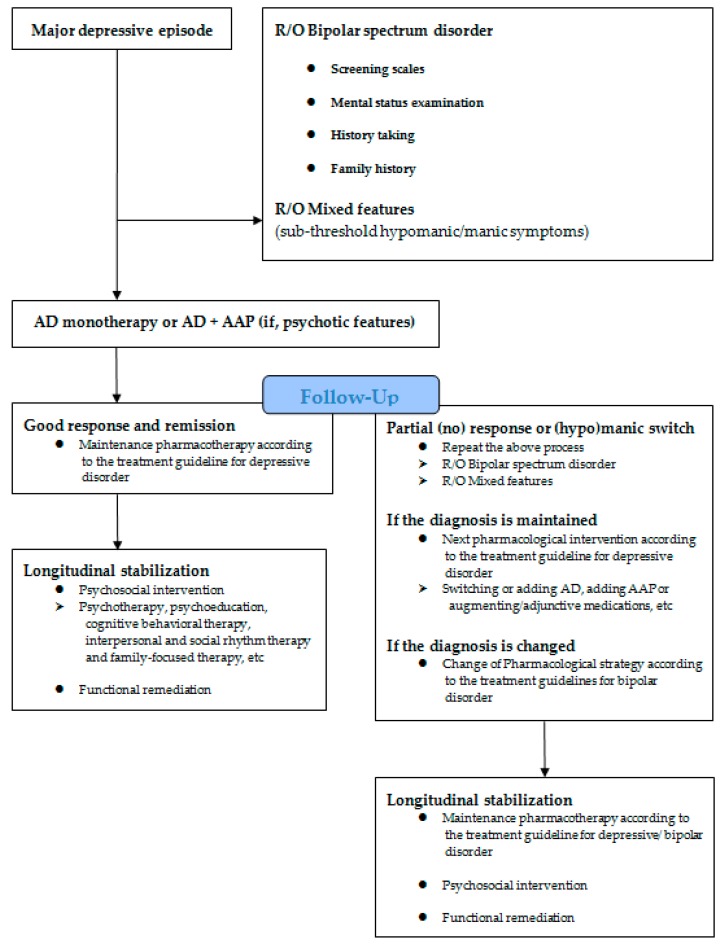
Treatment of major depressive episodes and the longitudinal stabilization of mood disorder. (Abbreviations: AD, antidepressant; AAP, atypical antipsychotics; MS, mood stabilizer).

In the past, most treatment strategies were based on the type of mood disorder and episodic polarity, which were differentiated early in treatment. However, recent diagnostic and treatment approaches have focused on transdiagnostic psychopathology, spectrum, and dimensional classifications. The process reflecting the tendency of these changes is shown in Figure 1. First, a syndromal concept, such as bipolar-spectrum disorder including bipolar I, II, and not otherwise specified (NOS) and mixed features presenting subthreshold hypo (manic) symptoms (although DSM-5 criteria for bipolar-spectrum disorder or mixed features specifier no longer met) should be checked at the beginning of MDE treatment. Careful assessment of bipolar symptoms using screening instruments (e.g., the hypomania checklist (HCL-32)), mood depression questionnaires (MDQs), screening assessment of depression-polarity (SAD-P)) or mental status examinations, patient history and clinical course, and family history is crucial to treatment decisions [22]. In addition, symptomatic (HAMD, MADRS, Young Mania Rating Scale (YMRS)) and functional (premorbid occupational and daily life functioning status) aspects must be evaluated [23].

## 3. ADs

### 3.1. AD Monotherapy

According to most clinical guidelines, AD monotherapy is recommended as the first-line treatment for non-psychotic MDEs without hypomanic or manic symptoms [1,3,24,25]. Newer AD agents, such as selective serotonin reuptake inhibitors (SSRIs) and serotonin-norepinephrine reuptake inhibitors (SNRIs) are preferred over older treatments [4]. These early stage MDEs usually require consideration of MDDs clinically first and follow the MDD treatment algorithms.

Studies evaluating prescription patterns indicate that ADs are prescribed for more than 50% of patients with bipolar depression [26,27]. Several randomized trials have found AD monotherapy to be an effective and safe treatment for bipolar depression; however, most of the studies were limited by small sample sizes, short durations, or lack of a control group [28,29,30]. The EMBOLDEN II trial found that paroxetine significantly improved HAMD scores, particularly in patients who had a good response and who had not used ADs previously [31].

AD monotherapy during the acute phase of a MDE must be administered carefully, as the response to ADs differs between MDD and BD patients. When ADs are used to treat bipolar depression, adverse effects may include mood switches, irritability, and agitation, even if not to the extent of mood switching; they can accelerate episode frequency or induce rapid cycling, and can increase the risk of suicide behaviors [8]. There is a limited role for ADs as an adjunctive therapy to MSs or AAPs for treating the acute phase of bipolar depression.

### 3.2. Efficacy and Safety of ADs

A meta-analysis of the efficacy and safety of ADs for bipolar depression revealed inconsistent results. One study indicated statistically significant overall efficacy of ADs in acute bipolar depression, while another reported that there was only a trend for higher response rates in patients treated with ADs [32,33].

Table 1 lists randomized controlled trials (RCTs) that evaluated ADs as a treatment for acute depression in BDs. Fluoxetine plus olanzapine (OFC) was the most effective treatment, while results for other ADs varied. Treatment with OFC was significantly more effective than olanzapine alone or the placebo in treatment of bipolar I depression, without increasing the risk of manic symptoms [34]. In another study, OFC was found more effective than lamotrigine for the treatment of bipolar I depression, although some increases in metabolic factors were observed [35]. Contrarily, paroxetine, bupropion, and agomelatine adjunctive therapies did not differ from the placebo or MS treatments [31,36,37]. 

Interestingly, the bupropion group showed a significantly lower risk of mood switch rates compared to other ADs (sertraline, venlafaxine, and desipramine); however, compared to other ADs, venlafaxine must be used cautiously [47,49,56]. Therefore, adjunctive treatments with SNRIs, such as venlafaxine or tricyclic antidepressants (TCAs), should be considered only after other ADs (e.g., bupropion, SSRIs) have been administered, and must be closely monitored due to an increased risk of mood switching and destabilization [8,33,57].

There is a lack of data regarding rapid cycling of ADs in RCTs. As part of the Systematic Treatment Enhancement Program for Bipolar Disorder (STEP-BD) study, AD continuation with rapid cycling was associated with worsened maintenance outcomes, especially for depressive morbidity, compared to the AD discontinuation group [58]. However, rapid cycling status may not be associated with a diminished response or greater depressive relapse during venlafaxine treatment relative to lithium monotherapy in bipolar II subjects [59]. Additional research is required in this area.

There is still a lack of evidence that the use of ADs for depression in BDs increases suicidal ideation and suicidal behavior, AD monotherapy of bipolar depression may entrain a course characterized by higher proneness to mood switches and suicidal behavior [60,61]. However, in some studies, patients with bipolar I and II disorders showed either no evidence of, or a significant reduction in, the risk of suicidal behavior during periods of AD exposure [62,63,64]. It should be noted that some suicidal subjects had unconfirmed mixed features [61,65,66].

### 3.3. Clinical Application of ADs

In clinical practice, most guidelines recommend combination therapy with MSs or AAPs, particularly lamotrigine and AAPs, rather than AD monotherapy for bipolar depression [4,5,7,67,68]. For mild to moderately severe cases, adjunctive AD therapy is often recommended as the second-line intervention, using SSRIs or bupropion combined with MSs or AAPs, particularly OFC. For moderate to severe cases, AD combination therapies are considered the first-line intervention.

Currently, there are no set guidelines for the dosage of ADs for bipolar depression. Tada et al. suggested that dose increments of adjunctive ADs might need to be considered for those receiving low doses who remain unresponsive [69]. Although there is a lack of evidence regarding the relationship between AD dose and mood switching in bipolar depression, use of adjunctive ADs may be associated with increased severity of manic symptoms in the STEP-BD [70].

### 3.4. ADs for Maintenance 

Clinically, long-term AD use for depression in BD patients is very common. However, their role in preventing depressive or manic episode relapse is doubtful. There seems to be a prophylactic effect on depressive episodes. In a RCT of long-term fluoxetine versus lithium monotherapy for bipolar II disorder, fluoxetine monotherapy provided superior relapse prevention compared to lithium monotherapy as a maintenance treatment, although the results for fluoxetine and the placebo were comparable [71]. In a STEP-BD study of ADs’ long-term effectiveness and safety, AD continuation with MSs trended toward less severe depressive symptoms (difference in DSM-IV depression criteria = −1.84, 95% CI −0.08–3.77) and mildly delayed depressive episode relapse, without increased manic symptoms (mean difference in DSM-IV mania criteria = +0.29, 95% CI −0.73–1.20), although a rapid cycling course predicted three times more depressive episodes with AD continuation (rapid cycling = 1.29, non-rapid cycling = 0.42 episodes/year, *p* = 0.04) [72].

However, in a meta-analysis of seven RCTs (350 bipolar depression patients), long-term treatment with MSs in combination with ADs yielded neither major protection from depression (RR = 0.84, 95% CI 0.56–1.27) nor a substantial increase in the risk of mania (RR = 1.37, 95% CI 0.81–2.33) [9]. In a study of venlafaxine continuation, there were no differences in relapse rates between venlafaxine and lithium (7.5% vs. 26.7%, *p* = 0.079), relapse hazard (*p* = 0.073), or time to relapse (*p* = 0.090), possibly due to the small sample size and lack of a placebo [29].

In conclusion, the efficacy of AD monotherapy in maintenance and in preventing recurrence are inconclusive. However, long-term AD use with anti-manic drugs in BDs may not increase the risk of relapse of mood disorder.

## 4. MSs

### 4.1. Lithium

In the clinical literature on the acute depressive phase of BDs, eight of nine controlled comparisons found lithium superior to the placebo in depressed bipolar patients [73]. For prophylactic treatment, the usefulness of lithium against bipolar depression was confirmed together with its specific effectiveness for suicide prevention [74]. These anti-suicidal effects are possibly due to reducing mood disorder relapse, but additional mechanisms must be considered since there is some evidence that lithium decreases aggression and, possibly, impulsivity [75]. However, some studies indicate that lithium may be less effective for depression than lamotrigine; lamotrigine may provide a spectrum of efficacy complementary to that of lithium [76,77].

Table 1 shows that AD adjunctive therapy in addition to lithium may be beneficial. This is particularly observed in patients who cannot tolerate high serum lithium levels or those who have symptoms that are refractory to the AD effects of lithium [44]. Among patients with delayed onset of therapeutic response and a high number of non-responders to ADs, there is firm evidence for lithium as an effective augmentation strategy, but only modest evidence that lithium accelerates the response to ADs in patients with depression [78].

From the viewpoint that the concept of disease gradually expands into the bipolar-spectrum, longitudinal follow-up found a positive response to lithium augmentation in treatment-resistant MDE-related bipolar-spectrum disorder [79]. This maintenance treatment, lithium monotherapy, was associated with a significantly reduced risk of both manic/mixed and depressed relapse [74]. Moreover, quetiapine combination significantly reduced the risk of relapse of manic/mixed and depressive episodes in bipolar illness compared to the placebo. The risk of early recurrence of BDs, especially of mania, was found to increase following discontinuation of lithium use and may exceed that predicted in the course of the untreated disorder [80].

Lithium is an essential form of treatment but must not be applied monotherapeutically for bipolar depression, particularly in the treatment of severe bipolar depression [81]. As a combination treatment, lithium effectively prevents relapse and is particularly useful in patients with a high risk of suicide.

### 4.2. Lamotrigine

There have been several long-term studies of the effects of lamotrigine in the treatment of bipolar depression. Two placebo-controlled 18-month trials of lamotrigine indicated that it was effective against depression and mania, with more robust activity against depression [77]. In a long-term study involving 124 patients with bipolar depression receiving lithium, addition of lamotrigine was significantly more effective than the placebo during the acute phase, and continued to be beneficial compared to the placebo during the maintenance phase [82]. However, the effects of lamotrigine on the acute phase of bipolar depression are diverse and need to be studied further. In a naturalistic, open-label study of patients with bipolar II disorder, treatment of acute depression with lamotrigine or lithium showed comparable response and remission rates [83]. Another RCT did not demonstrate efficacy of lamotrigine monotherapy as an acute treatment, although it was well tolerated [84]. A RCT of the acute phase, comparing OFC to lamotrigine, showed that OFC-treated patients showed significant improvement compared to lamotrigine-treated patients in Clinical Global Impression—Improvement (CGI-I) response and time to response, although the OFC treatment had undesirable side effects such as greater weight gain and some elevated metabolic factors compared to lamotrigine [35]. Most guidelines recommend lamotrigine as the first-line treatment for mild to moderate bipolar depression [4,5,7,67,68,85]. Additionally, lamotrigine monotherapy or combined with lithium or AAPs is generally recommended.

For maintenance treatment, it has been reported that lamotrigine is effective in the prevention of depression, particularly the combination of lithium and lamotrigine [86]. However, the overall pool effect was only modest, although the advantage over the placebo was larger in more severely depressed patients [87].

### 4.3. Valproate/Carbamazepine

There have been several meta-studies related to treatment of the acute phase of bipolar depression with valproate and carbamazepine. One study on valproate showed that patients’ responses to, and remission with, valproate were significantly greater than with the placebo [88]. According to another study, valproate and carbamazepine also produced favorable response rates compared to the placebo group, and the number needed to treat (NNT) was also low (valproate 4.4 and carbamazepine 3.4) [89]. However, because the sample size of both studies was small (*N* = 140 and *N* = 142, respectively), more research is needed to make a definitive conclusion.

For maintenance treatment, some research suggests that valproate may prevent depressive episodes. Valproate and carbamazepine may be effective for bipolar depression, leading to decreased worsening of depressive symptoms and a reduced probability of depressive relapse [74,90]. A pilot study reported that a combination of lithium and valproate significantly reduced the chances of relapse or recurrence, although it was more likely to cause moderate to severe adverse side effects [91]. A small RCT of carbamazepine concluded that it was as effective as lithium in the prophylaxis of bipolar affective disorder [92]. However, some studies have shown that the prophylactic effect of carbamazepine is relatively low compared to other MSs or lithium, and that it may be better for broader spectrum disorders, such as bipolar II or bipolar not otherwise specified rather than lithium [93,94,95,96]. Additionally, lithium combination treatment, rather than carbamazepine monotherapy, has been shown to help prevent new episodes of relapse [97].

In conclusion, there is little evidence for the efficacy of valproate or carbamazepine for acute bipolar depression, even though they are the most frequently prescribed MSs for BDs. To date, valproate and carbamazepine have been found to be less effective during acute-phase treatment of bipolar depression, maintenance treatment, and for depression relapse prevention compared to other MSs and lithium [98].

## 5. Novel Research Avenues

To date, there are no significant biomarkers for mood disorders. The role of clinical evaluation in distinguishing between MDDs and BDs is very important. As shown in the differential diagnosis process in Figure 1, the history of hypomanic symptoms and long-term follow-up are the most obvious ways to distinguish bipolarity. Thus, most patients with MDEs undergo early AD monotherapy. In the process of clinical examination and observation of pharmacological responses, if bipolarity or mixed features are suspected, or if there is a partial or no response to early AD monotherapy, the classes of ADs are appropriately modified and combined with lithium and MSs or AAPs. However, the trial and error process of psychiatric treatment can worsen symptoms such as mood switching and rapid cycling, and lead to suicidal behavior. Therefore, it is necessary to develop biomarkers that can distinguish between mood disorders biologically before initiating treatment. This requires an understanding of the pathophysiology associated with MDDs and BDs.

Recent findings suggest that molecular biology, genetic/epigenetic markers, neuroimaging, and inflammatory markers, oxidative stress, and mitochondrial dysfunction may be candidates to provide differential diagnosis and indicate drug responsiveness. The relationship of single nucleotide polymorphism (SNP) to AD responsiveness has been determined. Recently, gene-expression analysis has examined the involvement of miRNA in predisposing biological processes associated with antidepressant responses. miRNA in peripheral blood leukocytes may be a biomarker for MDDs, suicide, and cognitive function [99,100,101]. If genetic studies can predict a patient’s drug responsiveness, then the most appropriate drug can be selected as personalized medicine. In the case of mental illness, there are too many complex considerations involving social and psychological factors, and research on genetic pathophysiology has been lacking. However, in the case of other diseases, biological studies on pathophysiology and drug responses have been actively conducted and are already in the process of providing personalized drug treatments. Mood disorders, particularly MDEs, which are among the most common diseases in psychiatry will follow this trend.

For example, differences in fluoxetine response may be due to the influence of certain genes involved in fluoxetine transportation. Genetic variation in transport and remission, recovery, suicide risk, and other factors such as stress, family support, and other genetic factors are likely to be involved in MDD outcomes [102]. In addition, if venlafaxine is more effective in Val/Val patients than Met/Met carriers, the Catechol-O-methytransferase (COMT) Val/Met genetic polymorphism can be recommended as a biomarker for the prediction of venlafaxine efficacy in patients treated in psychiatric settings [103]. For MSs, evidence increasingly suggests that genetic factors play a strong role in the variation of response to lithium, and development of a method to predict individual responses to lithium could thereby accelerate recovery and reduce suffering and cost [104].

Neuroimaging, such as functional functional magnetic resonance imaging (fMRI) and positron emission tomography (PET), may reveal different responses in patients who respond well to a particular AD class or MS class, compared to those who do not respond. In PET of patients with unipolar depression, rostral anterior cingulate metabolism uniquely differentiated eventual treatment responders (hypermetabolism) from non-responders (hypometabolism) [105]. Increased glucose metabolism in cortico-limbic circuitry revealed by PET was related to successful paroxetine treatment response [106]. A recent fMRI study had better prognostic value than current practice based on clinical impressions [107,108]. Although various studies have examined the relationship between immune status, including various C-reactive protein or pro-inflammatory cytokines, and AD responsiveness, the results have been inconsistent. Peripheral blood inflammatory biomarkers may contribute to personalized treatment choice and improved AD outcomes [109].

The effects of oxidative stress on mood disorders are shown to result in a down-regulation of anti-oxidant reactions in the hippocampus, such as superoxide dismutase, catalase, glutathione peroxidase, glutathione-*S*-transferase, and other genes [110,111]. Accumulation of oxidative damage to mitochondria in brain might lead neuronal cellular death as a result of aggregation of oxidized protein, which may result in neurodegeneration [112]. In conclusion, to anticipate diagnostic differentiation of psychiatric illnesses and individual responses to classes of medication, and to realize personalized medicine, an understanding of the pathophysiology of depression and the development of simple and accessible biomarkers are required. To date, research on various basic sciences, such as genetic/epigenetic, proteomic, neuroimaging, and inflammatory factors, has been underway, and some candidate approaches have been suggested. However, no definite biomarker has yet been found and further studies are needed.

## 6. Conclusions

Historically, bisection of mood disorders into MDDs and BDs has been relatively rapid. Additionally, the concept of bipolar spectrum and the proportion of shared features between MDDs and BDs has led to increasingly divergent diagnoses. Under these circumstances, cautious pharmacological treatment must be administered using ADs and MSs in MDEs and for long-term stability of BDs. For initial treatment of MDEs, screening tools (e.g., HCL-32, MDQ, SAD-P, YMRS), specific symptom evaluation through mental status examination, and careful history taking should be performed to distinguish bipolarity from mixed features for which AD monotherapy may represent a risk. Depending on the therapeutic response, the existing guidelines and the results of empirical research findings can be followed. Currently, AD monotherapy should be avoided in patients strongly suspected as having bipolar depression or MDDs with mixed features. In these patients, a combination or adjunctive treatment with MSs or AAPs is recommended when it is considered beneficial to use ADs. MS efficacy studies on bipolar depression suggest that lamotrigine is the most reliable; lithium is expected to have modest effects, and evidence is still lacking regarding the efficacy of valproate and carbamazepine. To prevent long-term episodic relapse in BDs, MSs may also be recommended, either alone or in combination with other MSs and AAPs. Despite these therapeutic efforts, mood disorders are chronic psychiatric illnesses that are frequently recurrent and difficult to treat. There is a continuing need for novel research and further development of these drugs. Recently, radical progress has been achieved in research into pathophysiology and biomarkers in the fields of molecular biology, genetic/epigenetics, and neuroimaging. There is also an opportunity to study novel drug mechanisms through the rediscovery of fast-acting drugs, including ketamine. Through this process, it is anticipated that research will develop in the direction of discovering potential targets for new drugs and applying them to personalized treatments.

## Figures and Tables

**Table 1:** Randomized controlled trials of antidepressant use in acute phase in bipolar depression.

Study	Number of Bipolar Subjects (Type)	Duration (Weeks)	Antidepressant vs. Comparators (Doses, mg/d)	Primary Outcome Evaluation	Efficacy of Responders/Cases	Mood Switching
TCAs						
Bochetta et al., 1993 [38]	30 (BP I & II)	4	Amitriptyline 55 ± 10 vs. l-sulpiride 62 ± 13; add-on lithium	Response rate (≥50% reduction from baseline HAMD)	Amitriptyline 86% vs. l-sulpiride 93%; no significant differences	Only l-sulpiride 1 patients
Agosti and Steward, 2007 [39]	70 (BP II)	6	Imipramine 50–300 vs. Phenelzine 15–90 vs. Placebo	Responders (CGI-I 1 or 2)	Imipramine 56.5% vs. Phenelzine 52% vs. Placebo 22.7%; no comparison within BP, only BP vs. UP comparison	None
MAO inhibitors vs. TCAs						
Himmelhoch et al., 1991 [40]	56 (BP I & II)	6	Tranylcypromine 20–60 vs. Imipramine 100–300	CGI score of +2 or +3 for at least 2 weeks	Tranylcypromine 81% vs. Imipramine 48%; Tranylcypromine > imipramine	Tranylcypromine 12% vs. Imipramine 24%
Thase et al., 1992 [41]	16 (BP I & II)	6	Tranylcypromine >30 vs. Imipramine >150; Crossover study, vice versa in those non-responding in the initial study(101)	Unclear (BDI; HAM-D; Pittsburgh reversed vegetative symptom scale)	75% for Imipramine to Tranylcypromine vs. 25% for Tranylcypromine to Imipramine; no mention of significance	1/4 patients in imipramine (25%)
Silverstone, 2001 [42]	156 (BP I & II)	8	Moclobemide 450–750 vs. Imipramine 150–250	Change from baseline HAMD	Moclobemide 9.9% vs. Imipramine 13.0%; no significant difference	Moclobemide 3.7% vs. Imipramine 11%
SSRIs vs. placebo						
Cohn et al., 1989 [43]	89 (BP I & II)	6	Fluoxetine 20 to 80 vs. Imipramine 75 to 300 vs. Placebo	Response rate (>50% reduction from baseline HAMD)	Fluoxetine 86% vs. Imipraime 57% vs. Placebo 38%; Fluoxetine > placebo, fluoxetine > imipramine	Fluoxetine 0% vs. Imipramine 6.7% vs. Placebo 3.4%
Nemeroff et al., 2001 [44]	117 (BP I & II)	10	Paroxetine 20–50 vs. Imipramine 50–300 vs. Placebo; add-on lithium	Change from baseline HAMD and CGI-I	Paroxetine 45.5% vs. Imipramine 38.9% vs. Placebo 34.9%; no significant difference	Paroxetine 0% vs. Imipramine 7.7% vs. Placebo: 2.3%
Tohen et al., 2003 [34]	833 (BP I)	8	Fluoxetine 25–50 vs. OFC (olanzapine 6–12) vs. Placebo	Change from baseline MADRS	OFC −18.5 vs. Olanzapine −15.0 vs. Placebo −11.9; OFC > placebo, OFC > olanzapine	OFC 6.4% vs. Olanzapine 5.7% vs. Placebo 6.7% ;no significant difference
Sachs et al., 2007 [36]	366 (BP I & II)	26	Paroxetine 20–40 vs. bupropion 150–300 vs. Placebo; add-on mood stabilizers	Durable recovery : At least eight consecutive weeks of euthymia	Antidepressants 23% vs. Placebo 27.3%; no significant difference	Antidepressants 10.1% vs. Placebo 10.7%
McElroy et al., 2010 [31]	740 (BP I & II)	8	Paroxetine 20 vs. Quetiapine 300 or 600 vs. Placebo	Change from baseline MADRS	Paroxetine −13.76 vs. Quetiapine 300, −16.19 vs. Quetiapine 600, −16.31 vs. Placebo −12.60; both quetiapine > placebo, paroxetine > placebo	Paroxetine 10.7% vs. Quetiapine 300, 2.1% vs. Quetiapine 600, 4.1% vs. Placebo 8.9%
Altshuler et al., 2017 [45]	142 (BP II)	16	Sertraline >100 vs. Lithium >900 vs. Combination of both drugs	Switch to hypomania or mania	Sertraline 73.3% vs. Lithium 67.4% vs. Combination of both drugs 47.9%; no significant difference	Sertraline 17.8% vs. Lithium 14.3% vs. Combination of both drugs 10.4%; no significant difference
SSRIs vs. other drugs						
Young et al., 2000 [46]	27 (BP I & II)	6	Paroxetine 36 (mean) vs. Lithium 1300 (mean) or Divalproex 1200 (mean); add-on lithium or divalproex	Change from baseline HAMD, YMRS, and GAF	For HAMD, paroxetine + lithium or divalproex > lithium or divalproex; for YMRS, no difference; for GAF, paroxetine+lithium or divalproex > lithium or divalproex	Paroxetine was not associated with the emergence of manic symptoms
Vieta et al., 2002 [47]	60 (BP I & II)	6	Paroxetine: 20–60 vs. Venlafaxine: 75–450	Change from baseline HAMD	Paroxetine: 43% vs. Venlafaxine: 48%; venlafaxine > paroxetine	Paroxetine 3% vs. Venlafaxine 13%
Shelton et al., 2004 [48]	30 (BP I & II)	12	Paroxetine 35.0 ± 21.2 vs., Risperidone 2.15 ± 1.2 vs. Combination of both drugs; add-on mood stabilizers	Change from baseline HAMD	Paroxetine 5.6 ± 6.5vs, Risperidone 5.2 ± 8.7vs. Combination of both drugs 6.3 ± 6.5; no significant difference	None
Post et al., 2006 [49]	184 (BP I & II)	10	Sertraline 50–200 vs. Bupropion 75–450 vs. Venlafaxine 37.5–375; add-on mood stabilizers	Response ( ≥50% improvement in IDS score or a decrease in the CGI-BP depression score of ≥ 2)	Bupropion 49% vs. Sertraline 53% vs. Venlafaxine 51%; no significant difference	Bupropion 14%, Sertraline 16% Venlafaxine 31%; venlafaxine > bupropion or sertraline
Schaffer et al., 2006 [50]	20 (BP I & II)	12	Ciralopram 10–30 vs. Lamotrigine 50–200 (no divalproex) and 25–100 (with divalproex); add-on mood stabilizers	Change from baseline MADRS	Ciralopram −14.2 vs. Lamotrigine −13.3; no significant difference	Ciralopram 1 patient vs. Lamotrigine 1 patients; no significant difference
Brown et al., 2006 [35]	410 (BP I)	7	Fluoxetine (OFC) 25–50 vs. Lamotrigine 200	Change from baseline CGI-S	Fluoxetine (OFC) −1.43 vs. Lamotrigine −1.18; OFC > lamotrigine	Fluoxetine (OFC) 4.0% vs. Lamotrigine 5.2%; no significant difference
Altshuler et al., 2017 [45]	142 (BP II)	16	Sertraline >100 vs. Lithium >900 vs. Combination of both drugs	Switch to hypomania or mania	Sertraline 73.3% vs. Lithium 67.4% vs. Combination of both drugs 47.9%; no significant difference	Sertraline 17.8% vs. Lithium 14.3% vs. Combination of both drugs 10.4%; no significant difference
SNRIs						
Amsterdam, 1998 [51]	17 (BP II)	6	Venlafaxine 37.5–225	Change from baseline HAMD	Venlafaxine 10 ± 8; no comparison within BP, only BP vs. UP comparison	None
Amsterdam, 2000 [52]	15 (BP II)	6	Venlafaxine 37.5–225	Response rate (≥50% reduction from baseline HAMD)	Venlafaxine 63%; no comparison within BP, only BP vs. UP comparison	None
Amsterdam, 2016 [53]	129 (BP II)	12	Venlafaxine 37.5–75 vs. Lithium 300–600mg	Response rate (>50% reduction from baseline HAMD plus final CGI-S)	Venlafaxine 67.7% vs. Lithium 34.4%; venlafaxine > lithium	None
NDRIs						
Sachs et al., 1994 [54]	15 (BP I & II)	8	Bupropion 358 ± 62 vs. Desipramine 140 ± 46	Response rate (>50% reduction from baseline HAMD)	Bupropion 63% vs. Desipramine 71%; no significant difference	Bupropion 11% vs. Desipramine 50%; Desipramine > bupropion
Grossman et al., 1999 [55]	16 (BP I)	6	Bupropion 450 vs. Idazoxan 240; add-on lithium	Change from baseline HAMD	Bupropion −1.54 vs. Idazoxan −1.06; no significant difference	No mention
Agomelatine						
Yatham et al., 2016 [37]	344 (BP I)	8	Agomelatine 25–50 vs. Placebo; add on lithium or valproate	Change from baseline MADRS	Agomelatine −15.4 vs. Placebo −15.2; no significant difference	Agomelatine 4.1% vs. Placebo 3.5%

(Abbreviations: BDI, Beck Depression Inventory; HAMD, ; CGI-I, clinical global impression severity-improvement; MADRS, Montgomery-Asberg depression rating scale; OFC olanzapine-fluoxetine combination; IDS, Inventory of depressive symptomatology; CGI-BP, clinical global impression-bipolar version, YMRS, young mania rating scale; GAF, global assessment of functioning scale).
